# Image Deblurring Using Multi-Stream Bottom-Top-Bottom Attention Network and Global Information-Based Fusion and Reconstruction Network

**DOI:** 10.3390/s20133724

**Published:** 2020-07-03

**Authors:** Quan Zhou, Mingyue Ding, Xuming Zhang

**Affiliations:** Department of Biomedical Engineering, School of Life Science and Technology, Ministry of Education Key Laboratory of Molecular Biophysics, Huazhong University of Science and Technology, No 1037, Luoyu Road, Wuhan 430074, China; zhoudachuan@hust.edu.cn (Q.Z.); myding@hust.edu.cn (M.D.)

**Keywords:** image deblurring, Gaussian blur, deep learning, multi-stream bottom-top-bottom attention network, fusion network, reconstruction network

## Abstract

Image deblurring has been a challenging ill-posed problem in computer vision. Gaussian blur is a common model for image and signal degradation. The deep learning-based deblurring methods have attracted much attention due to their advantages over the traditional methods relying on hand-designed features. However, the existing deep learning-based deblurring techniques still cannot perform well in restoring the fine details and reconstructing the sharp edges. To address this issue, we have designed an effective end-to-end deep learning-based non-blind image deblurring algorithm. In the proposed method, a multi-stream bottom-top-bottom attention network (MBANet) with the encoder-to-decoder structure is designed to integrate low-level cues and high-level semantic information, which can facilitate extracting image features more effectively and improve the computational efficiency of the network. Moreover, the MBANet adopts a coarse-to-fine multi-scale strategy to process the input images to improve image deblurring performance. Furthermore, the global information-based fusion and reconstruction network is proposed to fuse multi-scale output maps to improve the global spatial information and recurrently refine the output deblurred image. The experiments were done on the public GoPro dataset and the realistic and dynamic scenes (REDS) dataset to evaluate the effectiveness and robustness of the proposed method. The experimental results show that the proposed method generally outperforms some traditional deburring methods and deep learning-based state-of-the-art deblurring methods such as scale-recurrent network (SRN) and denoising prior driven deep neural network (DPDNN) in terms of such quantitative indexes as peak signal-to-noise ratio (PSNR) and structural similarity (SSIM) and human vision.

## 1. Introduction

Image blurring is one of the major reasons for image quality degradation. It poses a great challenge for understanding and analyzing the high-frequency components in the images [1]. Therefore, deblurring has been a hot research field in image restoration [2,3,4,5,6]. Image deblurring is the process of inferring the latent sharp images in the absence of degradation model information. The blur types can be roughly divided into two categories. The first one is motion blur. For example, camera shake during exposure will blur the captured image. The second one is defocus blur produced by such factors as atmospheric turbulence and aberrations in the optical system. In most situations of practical interest, Gaussian blur is ubiquitous in optical imaging systems. Gaussian blur removal is highly significant in many applications; thus, it has attracted much attention in the field of image deblurring.

Many existing deblurring methods generally describe the blur process as

(1) $$B=I^{o}⊗k+n$$

where ⊗ denotes the 2D convolutional operator, B is the blurry input image, $I^{o}$ is the sharp image, k is the blur kernel, and n is the noise.

To find the sharp image in Equation (1) is an ill-posed problem. Solutions to this ill-posed problem can be divided into regularization methods and iterative schemes via updating and stopping rules. In more detail, the image deblurring methods include the optimization-based and the learning-based methods. Optimization-based algorithms firstly estimate the blur kernel and then realize non-blind deconvolution by using various image priors such as L0 gradients [7], sparse gradients [8,9,10], normalized sparsity [11], and patch priors [12] to regularize the solution space. For example, the nonlocally centralized spare representation (NCSR) [13] model has been presented to improve the performance of sparse representation-based image restoration by using the nonlocal self-similarity to estimate the sparse coding coefficients. Expected patch log likelihood (EPLL) [14] uses the regularization model based on the statistics of image patches for whole-image restoration. The block matching 3D filtering (BM3D) [15] method utilizes the formalized image modeling to realize the iterative deblurring. Michaeli and Irani [16] used self-similarity as an image prior to addressing the deblurring problem. Shan et al. [17] used a piecewise function to fit the gradient distribution of multiple natural images and then iteratively estimated the blur kernel function and the restored image. Pan et al. [18] presented the dark channel prior to image deblurring. This method can perform well in deblurring both natural images and text, face, and low-illumination images under the premise that the dark pixels can be extracted from an image. Chen et al. [19] proposed a deblurring method based on local maximum gradient prior and an effective optimization scheme. Yan et al. [20] proposed a novel image prior named extreme channels prior (ECP) by combining the bright channel prior with the dark channel one to improve the deblurred results. Although these methods have demonstrated good performance in some cases, they involve complicated computation and heuristic parameter-tuning [21]. Meanwhile, due to the utilization of hand-crafted priors that are generally designed based on the limited observations, these methods are not robust enough and may be ineffective for deblurring Gaussian blurred images with the complicated features.

With regard to the learning-based deblurring methods, deep learning has become very popular in the field of image deblurring. Up to now, the different deep learning models, such as generative adversarial network (GAN) and convolutional neural networks (CNN) (e.g., attentional convolutional network [22]) have been proposed. These deep learning-based deblurring methods can be roughly divided into three categories. The first one is to use the CNN to predict the blur kernel and then adopt the traditional non-blind deconvolution methods to restore the images. Sun et al. [23] proposed to predict the probabilistic distribution of motion blur for each patch using the CNN, and a deblurring model using the patch prior was adopted to remove motion blur. Yan et al. [24] used two neural networks to identify the blur type and then estimate its parameters, respectively. Cheng et al. [25] introduced the hand-crafted priors and the implicit deep priors trained by the CNNs as the regularization for the kernel estimation and used half-quadratic splitting optimization for image deconvolution. The performance of these methods greatly depends on the estimation accuracy of blur kernel. The second kind is to directly learn end-to-end mapping to output the deblurred results [26,27,28]. Tao et al. [21] presented a multi-scale recurrent network (SRN) that directly deblurs the wild images by combining the multi-scale images. Zhang et al. [29] presented the GAN-based image deblurring method in which spatial information and temporal information were combined as a generator. Wu et al. [30] presented a dual attention structure to dynamically aggregate the temporal cues for deblurring. These methods generally cannot handle serious image blurring effectively. The last kind is to use the model-driven deep learning methods for image deblurring. Dong et al. [31] presented an iterative deblurring method based on the denoising prior driven deep neural network (DPDNN). Distinctively, the alternating direction method of multipliers (ADMM) [32] technique is used to convert the constrained optimization problem into sub-problems, and the iterative process is unfolded into a deep neural network, which is composed of multiple denoiser modules for exploiting the multi-scale redundancies of natural images and back-projection modules for ensuring the observation consistencies.

Despite the advantage of the deep learning-based deblurring methods over the traditional ones, they still cannot perform well in restoring fine details and reconstructing sharp edges. In particular, small-scale details in the blurred image may be damaged seriously by the deblurring method in the case of the serious image blurring. To address this issue, we proposed two deep learning models to produce the deblurred image with the image details preserved well. For one, we proposed the multi-stream bottom–top–bottom attention network (MBANet) to extract image features at different scales using the multi-scale scheme. In the proposed MBANet, the multi-scale components of original images are firstly extracted and fed into the encoder–decoder ResBlock network simultaneously, which integrate high-level semantic information in the data stream with low-level spatial and channel-wise information. Then, the multi-scale output maps are produced by the multi-stream network with the multi-scale structure, which exploits the significant influence of multi-scales on Gaussian blur. On the other hand, to realize the effective fusion of output maps produced by the MBANet and produce the satisfactory deblurred image, the global information-based fusion and reconstruction network (GIFRNet) was proposed. This network includes the two sub-networks, i.e., the global information-based fusion network (GIFNet) and the global information-based reconstruction network (GIRNet). The GIFRNet integrates multi-scale output maps to greatly improve the global spatial information and continuously refine the outputs of GIFNet to compensate for the unwanted loss of image details in the deblurred image, especially the small-scale ones. Experiments were done on two different dataset to demonstrate the effectiveness and superiority of the proposed method to the state-of-the-art deblurring methods.

The main contributions of this study are summarized as follows:

1. We proposed a novel MBANet by combining the advantages of encoder-decoder structure and attention mechanisms to extract multi-scale and multi-level features effectively, thereby providing a solid foundation for recovering the fine details and reconstructing the sharp edges from the blurry images.
2. The proposed global information-based fusion network can merge the channel-wise and spatial information encoded in the bottom–top data stream at each scale effectively. Moreover, the proposed reconstruction network based on the fused global information can gradually refine the outputs of the GIFNet to finally produce the satisfactory deblurred images.
3. A new loss function was designed by combining the perceptual loss with the mean squared error to accelerate the gradient descent of the network, thereby leading to effective network training.

The remainder of the paper is organized as follows. In Section 2, we present the details of MBANet and the GIFRNet. In Section 3, the experimental results are provided. Finally, conclusions and future research directions are given in Section 4.

## 2. The Structure of the Proposed Networks

**Figure 1:**
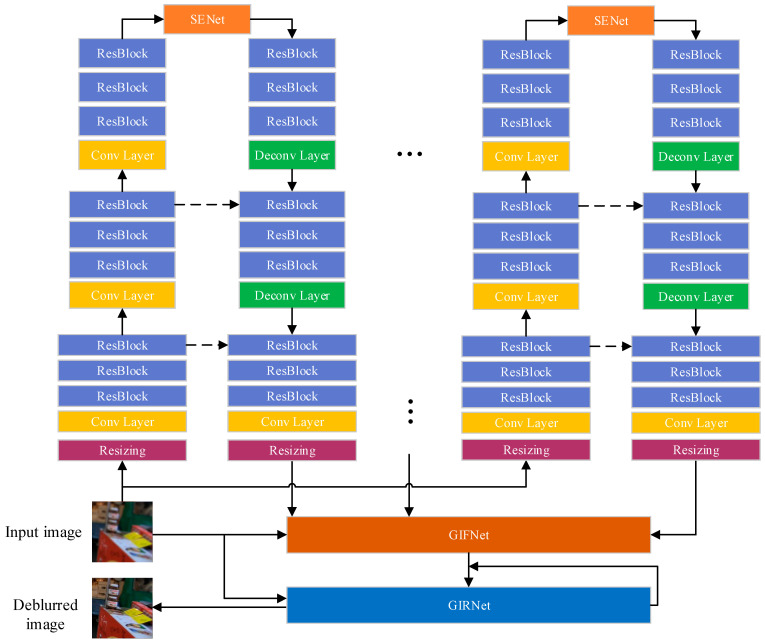
The flowchart of the proposed method. Each colored box is considered to be a feature block, and the arrows between blocks represent the information stream.

The architecture of the proposed networks is illustrated in Figure 1. As shown in Figure 1, the multi-scale images generated by the resizing operation are firstly fed into the MBANet, which combines a modified encoder-decoder ResBlock network with an attention mechanism. Then, the integration of backward and forward information is performed by the skipping connection. Based on the global information, the squeeze-and-excitation network (SENet) [33] is introduced here to implement the adaptive calibration of the channel-wise feature, highlight the important features in the encoder, and pass them to the decoder. Following the decoder, the resizing operation is again adopted to ensure that the generated multi-scale maps have the same resolution as the original input image. Finally, the GIFRNet is utilized to integrate the global feature information at each scale and refine the output deblurred image. We will provide the detailed description of the construction of the MBANet and the GIFRNet as well as the process of model training.

### 2.1. MBANet

With regard to the well-established multi-scale networks, there are two main types of network structures. The first one is to process images at different scales separately, which lead to the non-utilization of information between different scales. The second one is to cascade and share the weights among different scales. The integration of features by way of cascading makes network training easier and more stable, but its effectiveness greatly depends on the accuracy at the first scale. In this work, we proposed a parallel bottom-top-bottom network structure and introduced spatial and channel attention mechanisms. As opposed to other multi-scale methods, our method uses multi-scale images as the inputs of the network, so that the errors introduced by the first scale can be prevented from transmission to other scales. In order to greatly reduce training difficulty and increase stability, the network weights is shared across different scales.

The proposed end-to-end MBANet can be viewed as a regression network. From the perspective of network structure, there are several advantages. Firstly, the network is deep enough to generate a large receptive field for extracting different levels of features. Secondly, the combination of spatial and channel-wise information can facilitate the utilization of the global information to highlight the important features. Finally, the network combines the low-level cues and the high-level semantic information to improve the fineness of the deblurred image.

#### 2.1.1. Structure of Bottom-Top-Bottom Attention Network

**Figure 2:**
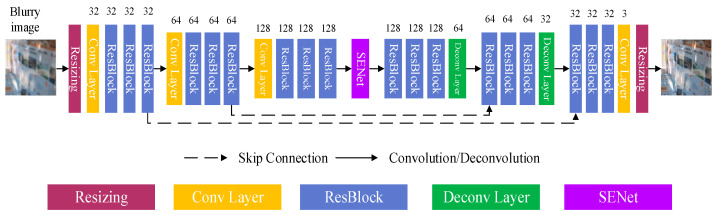
The architecture of the bottom–top–bottom attention network.

We chose the encoder-decoder model as the basic structure of the bottom-top-bottom [34] attention network and modified it to realize accurate feature extraction and combination. The encoder-decoder network [35] has a symmetric structure in which the input images are progressively transformed into feature maps with more channels and smaller sizes, and then the feature maps are transformed back to the resolution of the original input images. The skip connections between corresponding feature maps are the operations that combine the forward information, i.e., the extracted features in the encoder. It is easy to understand that the encoder is merely implemented using a series of convolutional (Conv) layers and the decoder contains several deconvolutional (Deconv) layers to further increase the network depth. However, the direct utilization of the existing encoder-decoder structure cannot ensure satisfactory performance. The reason is that we need to add more convolutional layers in the encoder/decoder blocks for deblurring tasks, which makes the network converge slowly. Moreover, it is difficult to obtain the spatial information from the middle feature maps with the small size for reconstruction. Hence, we chose to transform the encoder-decoder model into an encoder-decoder ResBlock network, as shown in Figure 2, which acts as our bottom-top backbone structure. Each backbone structure is comprised of one convolutional layer with a stride of two followed by several ResBlocks. The number of kernels at the convolutional layers is doubled for the successive backbone structure. Each ResBlock includes two convolutional layers with the same number of kernels. The network structure of the decoder is symmetric to that of the encoder. Therefore, the resolution of output images for the transformed encoder-decoder ResBlock network is the same as that of the original input images. It is highly significant to produce a large receptive field for extracting more feature information while making network training simpler. The encoder and the decoder are connected by the SENet, which can realize the information exchange between the channels, enhance the useful features, and suppress the features that are not important for the current task, thereby facilitating extraction of the key information in the feature maps at different channels with a reduction ratio of 64. For the SENet, its core operations include squeeze and excitation. The function of the squeeze operation is to encode the spatial features on a channel into a global feature, thereby allowing information from the global receptive field to be used by different layers. The aggregation is followed by an excitation operation, which adopts a simple self-gating mechanism to produce a collection of per-channel modulation weights. The learned channel weights are multiplied by the original input features to generate the channel feature $X_{c}^{n}$. It is given by

(2) $$Z_{c}^{n}=\frac{1}{W_{feature}\times H_{feature}}\sum_{i=1}^{W_{feature}}\sum_{j=1}^{H_{feature}}M_{c}^{n}\left(i,j\right)$$

(3) $$S_{c}^{n}=\sigma (W_{2}\theta (W_{1}Z_{c}^{n}))$$

(4) $$X_{c}^{n}=M_{c}^{n}\cdot S_{c}^{n}$$

where $Z_{c}^{n}$ represents the global spatial information of the *n*-th scale image at the *c*-th channel; $M_{c}^{n}$ is the feature map produced by the encoder; $W_{feature}$ and $H_{feature}$ are the width and height of $M_{c}^{n}$, respectively; $W_{1}\in {\mathbb{R}}^{\frac{c}{r_{se}}\times c}$ and $W_{2}\in {\mathbb{R}}^{c\times \frac{c}{r_{se}}}$ are the full connection operations with $r_{se}$ denoting the channel reduction ratio; θ means rectified linear unit (ReLu) activation [36]; σ denotes the sigmoid activation function; and $S_{c}^{n}$ is the channel-wise weight. From Equation (4), it can be seen that the channel-wise multiplication between $S_{c}^{n}$ and $M_{c}^{n}$ generates $X_{c}^{n}$.

#### 2.1.2. Structure of Multi-Stream Bottom-Top-Bottom Attention Network

Considering that Gaussian blur is sensitive to scale, we duplicated the designed bottom–top–bottom attention network in parallel. Specifically, the multi-scale versions of the input blurry images generated by the resizing operation are first encoded in the bottom–top stream. Then, for each input image, its scaled version $B^{n}$ replicates the first scale from the bottom layer to the top layer with a series of residual blocks, and a multi-stream output feature map $O^{n}$ (*n* = 1, 2, …, *Ns* with *Ns* denoting the number of scales) with the same resolution to $B^{n}$ is produced. Finally, these maps are resized to the same resolution to the original input image for the next network (see Figure 1).

### 2.2. GIFRNet

The GIFRNet consists of two sub-networks: GIFNet and GIRNet. The GIFNet integrates the maps generated by the MBANet to produce the feature map $R^{final}$. Then, the GIRNet gradually refines $R^{final}$ to produce the final deblurred image $I^{final}$.

#### 2.2.1. GIFNet

**Figure 3:**
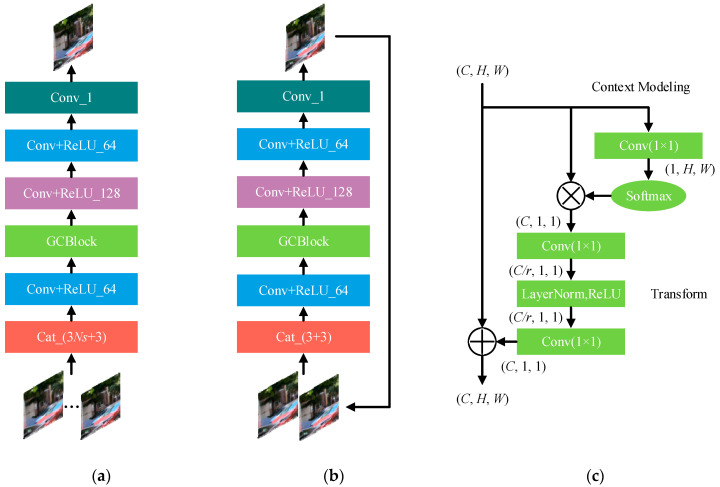
The architecture of the sub-network models. (**a**–**c**): GIFNet, GIRNet, and GCBlock, respectively. Here, (C,H,W) means a feature map with the number C of channels, height H, and width W; r denotes channel compression ratio; ⊗ represent matrix multiplication; ⊕ means broadcast element-wise addition.

The detailed structure of the GIFNet is shown in Figure 3a. As shown in Figure 3a, the dense spatial information of source images is combined with the multi-stream output maps, which are produced by the multi-stream network with the multi-scale structures to further increase the correlation of the concatenated maps. The original image B and the multi-stream output maps $\left(O^{1},O^{2},\ldots ,O^{Ns}\right)$ are firstly concatenated into a feature map $F^{0}$ with 3*Ns* + 3 channels. Then we add the global context block (GCBlock) [37], which can help establish a connection between two pixels with a certain distance to capture the spatial information in the feature images, thereby facilitating the fusion of the multi-stream information with the original image. The architecture of the GCBlock is shown in Figure 3c. It includes the context modeling module and the transform. The context modeling module groups the features of all positions together via weighted averaging to extract the global information. In order to reduce the calculation complexity, the channel compression transform is adopted. Furthermore, the layer normalization (LayerNorm) normalizes the mean and variance of all summed inputs to the neurons in one layer to improve the training speed, accuracy, and robustness of the model. The output features are fed to a series of convolutional and ReLU layers. In the convolutional layers, the kernel size is set to 3 × 3, and the channel number is set to 64, 128, 64, and 3, respectively. A merged map $R^{final}$ with the same resolution as the original image is produced as the final output. The GIFNet can be expressed as

(5) $$F^{0}=cat(O^{1},O^{2},\ldots ,O^{Ns},B)$$

(6) $$F^{1}=GCBlock(F^{0})$$

(7) $$F^{t}=\mathrm{ReLu}(W_{t}\cdot F^{t-1}+b_{t})$$

(8) $$R^{final}=W_{T}\cdot F^{T-1}+b_{T}$$

where cat denotes the concatenation operator; $F^{1}$ means the feature information produced by the GCBlock with the channel compression ratio set to 2; $F^{t}$ denotes the feature image generated at the *t*-th (*t* = 2, 3, …, *T*) convolutional layer; $W_{t}$ and $b_{t}$ represent the convolutional filter and bias of *t*-th convolutional layer, respectively; and the GIFNet fully utilizes the dense spatial information of the original image and nonlinearly fuses the multi-stream output maps.

#### 2.2.2. GIRNet

Although the GIFNet can integrate the feature maps at different scales effectively and improve spatial correlation by introducing the source image, errors may be introduced into the restored image. In order to address this issue, we introduced the GIRNet to further refine the output deblurred image. Figure 3b shows the network structure of the GIRNet, which has the same structure to the GIFNet but with different parameters. At each iteration, we feed both the original image and the input map through the GIRNet to produce the refined map, which in turn serves as the input map at the next iteration. The final restored image $I^{final}$ can be expressed as

(9) $$I^{final}=△(△(\cdot \cdot \cdot △(R^{final};B,W_{m},b_{m})\cdot \cdot \cdot ))$$

where $R^{final}$ is the preceding integrated map as the first input map. The operator △ means a recursive composition function. $W_{m}$ and $b_{m}$ represent the convolutional filter and the bias of the GIRNet, respectively. The final restored image is produced by continuously correcting its previous errors until the last iteration is implemented.

### 2.3. Loss

We used Euclidean loss and perceptual loss [38] as the total loss to measure the difference between the network output and the ground truth. Here, the Euclidean loss is the classic mean square error (MSE) loss. Unlike the pixel-level MSE loss, the perceptual loss utilizes high-dimensional features obtained from high-performance convolutional neural networks and can help restore more details for the deblurred images. The proposed total loss is defined as

(10) $$L_{MSE}={\left\|I^{final}-I^{o}\right\|}_{2}^{2}$$

(11) $$L_{c}={\left\|\phi_{k,l}(I^{final})-\phi_{k,l}(I^{o})\right\|}_{2}^{2}$$

(12) $$L_{total}=L_{MSE}+\lambda \cdot L_{c}$$

where λ is the parameter to balance $L_{MSE}$ and $L_{c}$, and $\phi_{k,l}$ denotes the feature extractor for extracting the image features at the *l*-th convolutional layer (after activation) before the *k*-th pooling layer in the VGG16 network pre-trained on the ImageNet dataset. The features extracted by $\phi_{k,l}$ are used to measure the difference between the high-level semantic information of images $I^{final}$ and $I^{o}$. The Adam optimizer [39] is adopted to minimize the cost function.

### 2.4. Model Training

To train the proposed networks, we used the realistic and dynamic scenes (REDS) dataset [40], in which each image was of size 720 × 1280. It was recorded by the GoPro HERO6 Black camera at 120 fps. There were 300 sequences in total, and each one included 100 pairs of sharp and blurry frames. We randomly selected 1120 sharp images as the ground truth for training and manually synthesized the blurry images by convolving the sharp images with Gaussian blur kernels of size 17 × 17. The standard deviations of the blur kernels were changed from 1.6 to 2.4 with steps of 0.2 to generate 5 blur kernels. The blurry images are sampled and randomly cropped into 256 × 256 image patches as training images. The ground-truth patches were generated in the same way. In total, 1,120,000 patches were generated for training.

We realized the proposed networks with TensorFlow 1.9.0. The proposed MBANet and GIFRNet were jointly trained on a Ubuntu 16.04 with an Intel I7-6950 CPU and 96G RAM. The NVIDIA 1080Ti GPU with CUDA 10.0 was used for acceleration. For the proposed networks, the initial learning rate was set to 0.0001, and it was exponentially decayed to 1 × 10^−6^ at 1000 epochs using power 0.3. At each iteration, we fixed the batch size to be 10. All trainable variables were initialized by Xavier [41]. The training took approximately 1.5 days.

## 3. Experimental Results and Discussion

In this section, we carried out experiments on the chosen REDS dataset and GoPro dataset [42] to determine the key parameters in the proposed networks and evaluate their performance. The testing data were generated in the same way as the training data. For each type of dataset, 10 images were randomly selected as testing images. To evaluate the deblurring performance of the proposed networks, we adopted such indexes as peak signal-to-noise ratio (PSNR) and structural similarity (SSIM) [43], which are defined as follows:

(13) $$\mathrm{PSNR}=10\log_{10}\left(\frac{255^{2}}{\frac{1}{W\cdot H}\sum_{i=1}^{W}\sum_{j=1}^{H}{\left(I^{final}\left(i,j\right)-I^{o}\left(i,j\right)\right)}^{2}}\right)$$

(14) $$\mathrm{SSIM}=\frac{\left(2{\bar{I}}^{final}{\bar{I}}^{o}+C_{1}\right)\left(2\delta_{I^{final}I^{o}}+C_{2}\right)}{\left({\left({\bar{I}}^{final}\right)}^{2}+{\left({\bar{I}}^{o}\right)}^{2}+\theta_{1}\right)\left(\delta_{I^{final}}^{2}+\delta_{I^{o}}^{2}+\theta_{2}\right)}$$

where W and H are the width and height, respectively, of the deblurred image $I^{final}$. $\delta_{I^{final}}$ and $\delta_{I^{o}}$ are the standard deviations of $I^{final}$ and $I^{o}$, respectively. ${\bar{I}}^{final}$ and ${\bar{I}}^{o}$ are the mean of $I^{final}$ and $I^{o}$, respectively; $\delta_{I^{final}I^{o}}$ is the covariance of $I^{final}$ and $I^{o}$. $C_{1}$ and $C_{2}$ are the small constants to stabilize SSIM.

### 3.1. Parameter Setting

As for the parameters in the proposed networks, they mainly include the number of scales (*Ns*) in the MBANet, the iterative times (*Tr*) in the GIRNet, the regularization parameter λ for balancing the content loss, and the perceptual loss and parameters *k* and *l* at the feature layer related to the perceptual loss. In the loss function of the proposed networks, the content loss and the perceptual loss are of the same order of magnitude. Following the selection method of parameters in the loss function in other deep learning models for image deblurring, we set λ=1 and *k* = *l* = 3. Here, we discuss how to determine the two parameters *Ns* and *Tr*.

#### 3.1.1. Number of Scales

**Figure 4:**
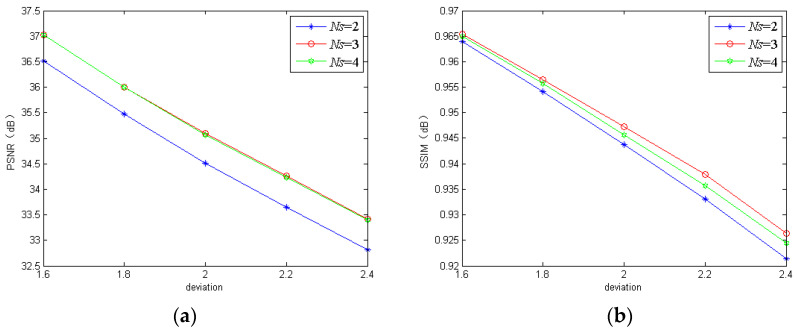
The peak signal-to-noise ratio (PSNR) and structural similarity (SSIM) of the proposed model using different numbers of scales on the realistic and dynamic scenes (REDS) dataset: (**a**) PSNR; (**b**) SSIM.

**Figure 5:**
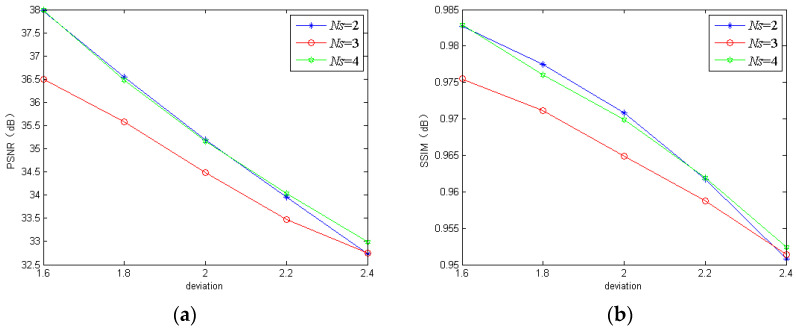
The PSNR and SSIM of the proposed model using different numbers of scales on the GoPro dataset: (**a**) PSNR; (**b**) SSIM.

As for the number of scales in the MBANet, it is easy to understand that too large an *Ns* value brings much calculation burden while too small a value is disadvantageous for extracting the effective features from the input images. Here, we trained the proposed model using three different scales for comparison to analyze the contributions of *Ns*. The PSNR and SSIM of the three models performed on the REDS dataset and GoPro dataset are shown in Figure 4 and Figure 5, respectively. Clearly, the three-scale model and the two-scale model could only perform well on the REDS dataset and GoPro dataset, respectively. However, the four-scale model could obtain better or competitive results on both the REDS dataset and GoPro dataset in terms of PSNR and SSIM. Therefore, we chose four scales as a compromise.

#### 3.1.2. Iterative Times

**Figure 6:**
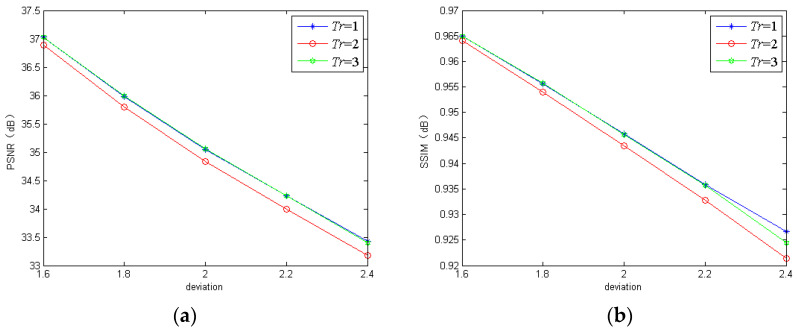
The PSNR and SSIM of the proposed model using different iterative times on the REDS dataset: (**a**) PSNR; (**b**) SSIM.

**Figure 7:**
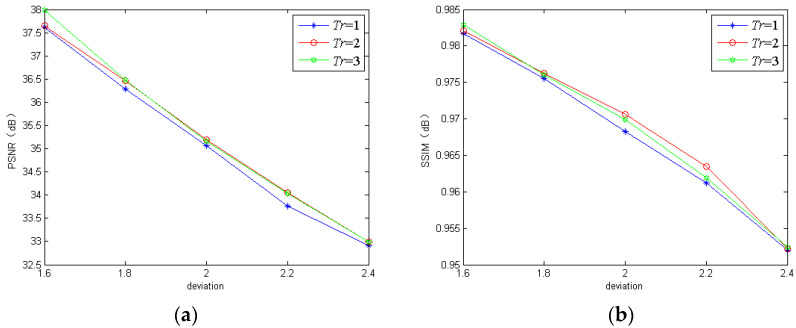
The PSNR and SSIM of the proposed model using different iterative times on the GoPro dataset: (**a**) PSNR; (**b**) SSIM.

For the iterative times *Tr* in the GIRNet, we found that too large a *Tr* increased the training time of the proposed networks greatly, while one that was too small could not ensure the satisfactory deblurring performance. To demonstrate the influence of *Tr*, we trained the proposed model using three different iterative times. The quantitative results are shown in Figure 6 and Figure 7. From Figure 6 and Figure 7, we can see that the proposed model with *Tr* = 2 performed the worst on the REDS dataset, and our model with *Tr* = 1 performed the worst on the GoPro dataset. By comparison, our model with *Tr* = 3 could achieve satisfactory performance on the two datasets. Based on the above analysis, we chose *Tr* = 3 in the proposed networks.

### 3.2. Comparison with Different Network Structures

**Figure 8:**
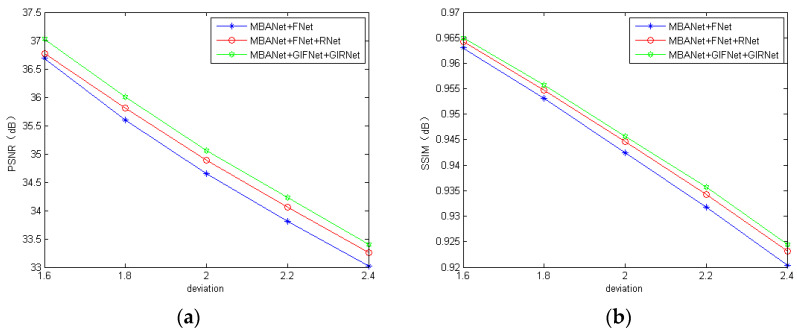
The PSNR and SSIM of different network models: (**a**) PSNR; (**b**) SSIM.

In order to verify the effectiveness of the structure in the proposed networks, we removed GCBlock from GIFNet and GIRNet. The resultant networks were named as fusion network (FNet) and reconstruction network (RNet), respectively. Here, we compare the three models, i.e., MBANet with FNet (MBANet + FNet), MBANet with FNet and RNet (MBANet + FNet + RNet), and MBANet with GIFNet and GIRNet (MBANet + GIFNet + GIRNet). We also used ten images chosen from the REDS dataset as the sharp images for testing. The quantitative results of PSNR and SSIM for the three models are shown in Figure 8. It can be seen from Figure 8 that MBANet + GIFNet + GIRNet achieved the best performance among the three models, which indeed indicates that the adoption of attention mechanism and reconstruction network structure improved the deblurring performance effectively.

### 3.3. Comparison with State-of-the-Art Deblurring Methods

To demonstrate the advantage of the proposed method, we compared it with five state-of-the-art image deblurring algorithms including three well-known model-based deblurring methods such as EPLL [14], NCSR [13], and ECP [20], and two deep learning methods such as SRN [21] and DPDNN [31]. For the compared algorithms, we firstly followed the parameter settings suggested by the authors and then tuned them to ensure the best results for the corresponding testing images based on the comprehensive consideration of PSNR and SSIM as well as subjective human vision. For fairness, unless noted otherwise, all experiments were conducted for the evaluated methods on the same dataset with the same parameter configuration.

#### 3.3.1. Comparison on REDS Dataset

Experiments were done on the color images in the REDS dataset. Table 1 and Table 2 list the PSNR and SSIM values of all evaluated methods. From Table 1, we can see that the deep learning methods outperformed the ECP, EPLL, and NCSR methods by providing significantly higher PSNR. Compared with the SRN and DPDNN methods, the proposed algorithm, on average, provided PSNR improvements by 0.42 dB and 0.27 dB, respectively. Table 2 shows that the SSIM values of the conventional model-based methods were lower than those of the deep learning methods. Meanwhile, the proposed method could provide slightly higher SSIM than the SRN and DPDNN methods.

**Figure 9:**
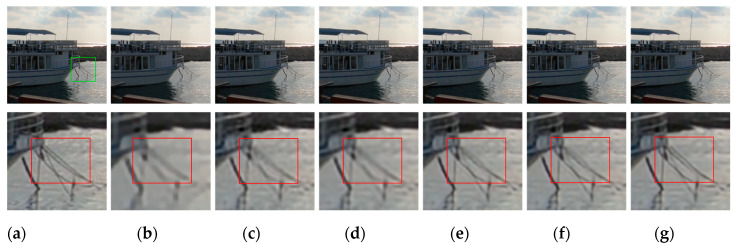
Deblurred results for test image with 17 × 17 Gaussian blur kernel of deviation 1.6. The first row shows the sharp image and deblurred images for the evaluated methods. The second row shows the enlarged views of the region marked with the green box in the original image and the deblurred images. (**a**) Original image [37]; (**b**) ECP; (**c**) EPLL; (**d**) NCSR; (**e**) SRN; (**f**) DPDNN; (**g**) proposed method.

**Figure 10:**
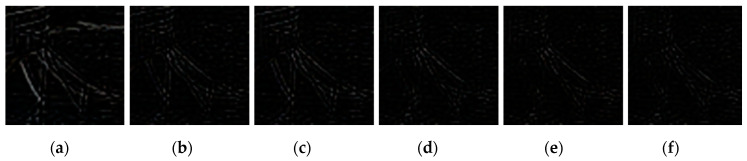
Difference images between the region marked with the green box in Figure 9a and its deblurred results. (**a**) ECP; (**b**) EPLL; (**c**) NCSR; (**d**) SRN; (**e**) DPDNN; (**f**) proposed method.

**Figure 11:**
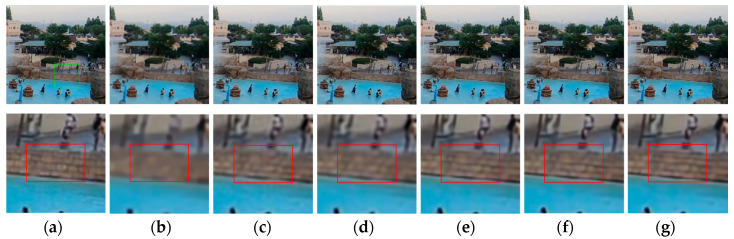
Deblurred results for test image with 17 × 17 Gaussian blur kernel of deviation 1.8. The first row shows the sharp image and deblurred images for the evaluated methods. The second row shows the enlarged views of the region marked with the green box in the original image and the deblurred images. (**a**) Original image [37]; (**b**) ECP; (**c**) EPLL; (**d**) NCSR; (**e**) SRN; (**f**) DPDNN; (**g**) proposed method.

**Figure 12:**
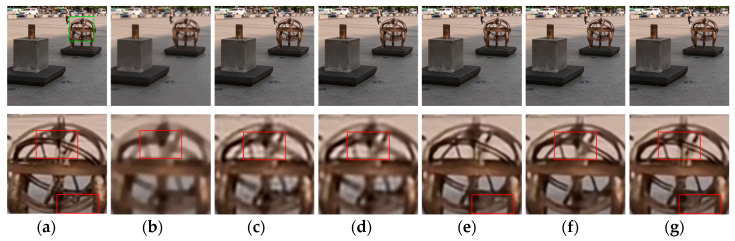
Deblurred results for test image with 17 × 17 Gaussian blur kernel of deviation 2.0. The first row shows the sharp image and deblurred images for the evaluated methods. The second row shows the enlarged views of the region marked with the green box in the original image and the deblurred images. (**a**) Original image [37]; (**b**) ECP; (**c**) EPLL; (**d**) NCSR; (**e**) SRN; (**f**) DPDNN; (**g**) proposed method.

**Figure 13:**
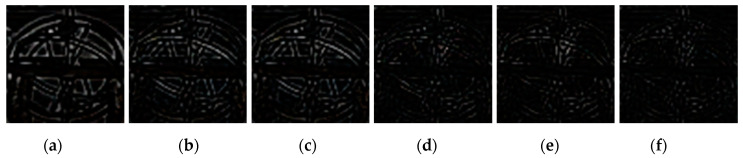
Difference images between the region marked with the green box in Figure 12a and its deblurred results. (**a**) ECP; (**b**) EPLL; (**c**) NCSR; (**d**) SRN; (**e**) DPDNN; (**f**) proposed method.

**Figure 14:**
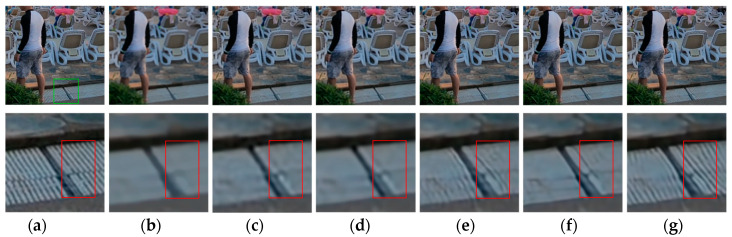
Deblurred results for test image with 17 × 17 Gaussian blur kernel of deviation 2.2. The first row shows the sharp image and deblurred images for the evaluated methods. The second row shows the enlarged views of the region marked with the green box in the original image and the deblurred images. (**a**) Original image [37]; (**b**) ECP; (**c**) EPLL; (**d**) NCSR; (**e**) SRN; (**f**) DPDNN; (**g**) proposed method.

**Figure 15:**
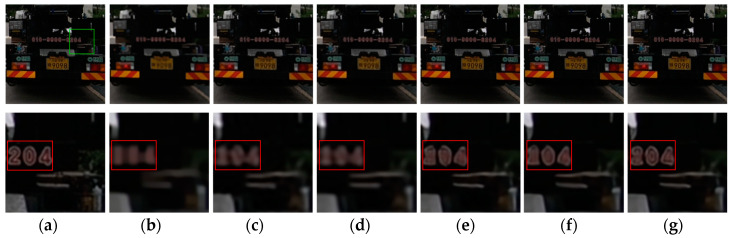
Deblurred results for test image with 17 × 17 Gaussian blur kernel of deviation 2.4. The first row shows the sharp image and deblurred images for the evaluated methods. The second row shows the enlarged views of the region marked with the green box in the original image and the deblurred images. (**a**) Original image [37]; (**b**) ECP; (**c**) EPLL; (**d**) NCSR; (**e**) SRN; (**f**) DPDNN; (**g**) proposed method.

**Figure 16:**
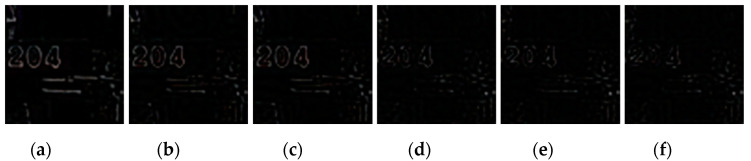
Difference images between the region marked with the green box in Figure 15a and its deblurred results. (**a**) ECP; (**b**) EPLL; (**c**) NCSR; (**d**) SRN; (**e**) DPDNN; (**f**) proposed method.

Figure 9, Figure 10, Figure 11, Figure 12, Figure 13, Figure 14, Figure 15 and Figure 16 show the deblurred results and the corresponding difference images of all evaluated methods. Here, we used the red boxes to indicate where the proposed method was visually superior to other state-of-the-art methods. As shown in Figure 9 and Figure 11, it can be seen that our proposed method could recover the thin lines in Figure 9 and the boundaries of stone steps in Figure 11 better than the other compared methods by providing more complete and sharper edges for them. It is shown in Figure 12 that the multi-scale deblurring approaches such as the proposed method and the SRN method gained an obvious advantage over other methods in recovering small-scale details such as the vertical thin iron rod marked with one red box. For example, the DPDNN method damaged the thin iron rod so seriously that it was almost invisible in the deblurred image while it was preserved significantly better by the SRN method and the proposed method. Furthermore, the proposed method outperformed the SRN method by providing a sharper restored result for the complex structures marked with another red box. As seen in Figure 14, the textures were so complicated and densely arranged that their restoration was very challenging. Clearly, the other compared methods led to the loss of textures in the deblurred images to some extent. However, the proposed method could still recover the textures effectively. Moreover, Figure 15 shows that in the case of large deviations of blur kernels, our method could preserve such image details as the number on the car more effectively than other evaluated methods. Furthermore, from the difference images between the original image and the deblurred images shown in Figure 10, Figure 13 and Figure 16, we can see that the traditional methods performed worse than the deep learning-based methods because there was too much structural information in their difference images. Compared with the SRN and DPDNN methods, the proposed method performed better in that it produced less obvious structural information, which further proves the superiority of the proposed method.

In order to further verify the robustness of the proposed method, we selected four different levels of Gaussian blur kernels with standard deviations ranging from 1.7 to 2.3 with a step of 0.2. These blurred images were not used as training sets for model training. The PSNR and SSIM values of all evaluated methods are listed in Table 3 and Table 4. It can be seen that the deep learning-based methods still performed better than the traditional methods. Compared with the SRN and DPDNN methods, the proposed method provided slightly higher SSIM values and it, on average, provided improvements of PSNR by 0.52 dB and 0.6 dB, respectively. The above comparison indeed demonstrated good robustness of the proposed method.

#### 3.3.2. Comparison with State-of-the-Art Methods on the GoPro Dataset

To further evaluate the performance of the proposed method, we used the GoPro dataset, which is different from the REDS dataset, to compare it with other deblurring methods. The quantitative results computed on the 10 images selected from this dataset are listed in Table 5 and Table 6. The results show that the deep learning-based algorithms still performed better than the traditional algorithms in PSNR and SSIM. The SRN performed worse than the DPDNN and the proposed method. Although the proposed method provided slightly lower PSNR and SSIM results than the DPDNN when the deviation was 2.4, it produced higher average PSNR and SSIM than the latter.

**Figure 17:**
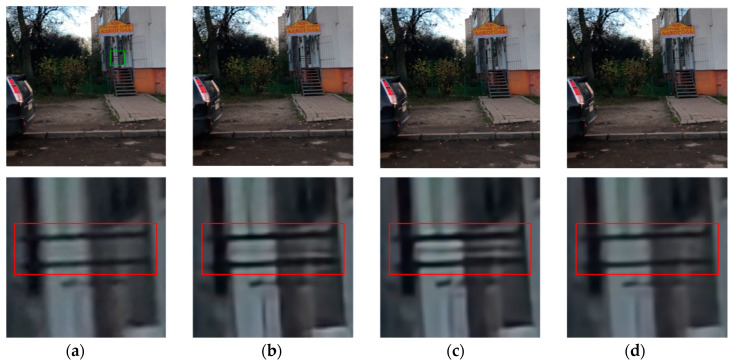
Deblurred results for three deep learning methods performed on the GoPro test image with 17 × 17 Gaussian blur kernel of deviation 2.2. The first row shows the sharp image and deblurred images by the evaluated methods. The second row shows the enlarged views of the region marked with green box in the original image and the deblurred images. (**a**) original image [39]; (**b**) SRN; (**c**) DPDNN; (**d**) proposed method.

Figure 17 shows the visual comparisons of the deblurred images for three deep learning-based deburring methods performed on the GoPro dataset, where the standard deviation of the Gaussian blur kernel in the test image was 2.2. From Figure 17, it can be seen that the SRN and the DPDNN produced the unwanted artefacts around the horizontal lines in the red box to different extents. By comparison, our method could generate more similar deblurred result as the unblurred sharp image without introducing the artefacts due to the combination of the features from the original image with the global context information. Indeed, the visual comparison demonstrated the superiority of the proposed method to other deep learning-based methods in recovering image details.

### 3.4. Discussion

In this work, we proposed the novel multi-stream bottom–top–bottom attention network as well as the global information-based fusion and construction network for restoring Gaussian blurred images. From the above experimental results, it can be seen that the proposed method gains an advantage over other compared methods in terms of deblurring performance and robustness. However, this work has some limitations. Firstly, the proposed networks are only used for the deblurring of Gaussian blurred images. Future work will be focused on extending our method to address other kinds of blur such as motion blur and the hybrid blur. Such extension will involve the modification of network structure such as the introduction of dense blocks. Secondly, the training of the proposed networks is very time-consuming. The model training time can be shortened by simplifying the network structure. Finally, the loss function we used is relatively simple, and thus there may be possibilities for further improvement of deblurred results. The effective structural preserving loss item can be explored and additionally introduced to address this problem.

## 4. Conclusions

In this paper, we proposed the novel multi-stream bottom–top–bottom attention network as well as the global information-based fusion and construction network for restoring Gaussian blurred images. Distinctively, the proposed network fully combines high-level semantics with low-level image features and channel-wise information to generate the deblurred images. Besides, the multi-stream mechanism fully takes advantage of multi-scale information, and this structure is easier to train by sharing the parameters. Additionally, the reconstruction network can further refine the restored image by combining the global context information. Experimental results show that the proposed method can provide better deblurring performance than other state-of-the-art algorithms both quantitatively and qualitatively.

## Figures and Tables

**Table 1:** The PSNR results of deblurred images for all evaluated methods.

Methods	Standard Deviation					Average
	1.6	1.8	2.0	2.2	2.4	
ECP	29.72	29.48	29.26	28.94	28.18	29.12
EPLL	33.25	32.29	31.45	30.73	30.12	31.57
NCSR	33.90	32.66	31.66	30.85	30.16	31.85
SRN	36.55	35.53	34.66	33.85	33.05	34.73
DPDNN	36.93	35.78	34.76	33.89	33.04	34.88
Proposed	37.03	36.00	35.05	34.23	33.40	35.14

**Table 2:** The SSIM results of the deblurred images for all evaluated methods.

Methods	Standard Deviation					Average
	1.6	1.8	2.0	2.2	2.4	
ECP	0.8905	0.8656	0.8483	0.8387	0.8171	0.8520
EPLL	0.9311	0.9168	0.9025	0.8882	0.8743	0.9026
NCSR	0.9363	0.9196	0.9038	0.8884	0.8736	0.9043
SRN	0.9622	0.9518	0.9417	0.9313	0.9198	0.9414
DPDNN	0.9643	0.9540	0.9431	0.9324	0.9211	0.9430
Proposed	0.9650	0.9557	0.9457	0.9357	0.9245	0.9453

**Table 3:** The PSNR results of deblurred images for all evaluated methods.

Methods	Standard Deviation				Average
	1.7	1.9	2.1	2.3	
ECP	29.55	29.40	29.07	28.36	29.10
EPLL	32.09	31.28	30.56	29.94	30.97
NCSR	33.24	32.15	31.24	30.49	31.78
SRN	35.00	34.34	33.63	32.91	33.97
DPDNN	34.99	34.13	33.61	32.81	33.89
Proposed	35.61	34.98	34.17	33.20	34.49

**Table 4:** The SSIM results of the deblurred images for all evaluated methods.

Methods	Standard Deviation				Average
	1.7	1.9	2.1	2.3	
ECP	0.8705	0.8575	0.8419	0.8217	0.8479
EPLL	0.9239	0.9095	0.8951	0.8810	0.9024
NCSR	0.9279	0.91157	0.8959	0.8810	0.9041
SRN	0.9497	0.9409	0.9312	0.9216	0.9359
DPDNN	0.9513	0.9409	0.9315	0.9213	0.9363
Proposed	0.9563	0.9478	0.9385	0.9293	0.9430

**Table 5:** The PSNR results of the deblurred images in the GoPro dataset.

Methods	Standard Deviation					Average
	1.6	1.8	2.0	2.2	2.4	
ECP	32.07	32.08	32.09	31.51	31.29	31.81
EPLL	32.24	31.52	30.68	29.96	29.36	30.75
NCSR	36.53	34.67	33.19	31.96	30.96	33.46
SRN	37.17	34.97	34.52	32.99	32.02	34.33
DPDNN	37.58	36.49	35.10	33.82	33.08	35.21
Proposed	37.99	36.48	35.16	34.04	32.99	35.33

**Table 6:** The SSIM results of the deblurred images in the GoPro dataset.

Methods	Standard Deviation					Average
	1.6	1.8	2.0	2.2	2.4	
ECP	0.8861	0.8588	0.8494	0.8297	0.8154	0.8479
EPLL	0.9611	0.9548	0.9458	0.9347	0.9228	0.9438
NCSR	0.9742	0.9636	0.9514	0.9377	0.9235	0.9501
SRN	0.9777	0.9687	0.9648	0.9529	0.9430	0.9614
DPDNN	0.9808	0.9760	0.9691	0.9598	0.9549	0.9681
Proposed	0.9829	0.9760	0.9699	0.9619	0.9524	0.9686

## References

[1] Flusser J., Farokhi S., Höschl C., Suk T., Zitová B., Pedone M. (2015). Recognition of Images Degraded by Gaussian Blur. IEEE Trans. Image Process..

[2] Li J., Liu Z. (2019). Ensemble Dictionary Learning for Single Image Deblurring via Low-Rank Regularization. Sensors.

[3] Liu D., Chen X., Shi C., Liu X. (2019). Star Image Prediction and Restoration under Dynamic Conditions. Sensors.

[4] Li J., Gong W., Li W. (2018). Combining Motion Compensation with Spatiotemporal Constraint for Video Deblurring. Sensors.

[5] Yang Z., Yang Z., Gui G. (2018). A Convex Constraint Variational Method for Restoring Blurred Images in the Presence of Alpha-Stable Noises. Sensors.

[6] Yang F., Huang Y., Luo Y., Li L., Li H. (2016). Robust Image Restoration for Motion Blur of Image Sensors. Sensors.

[7] Pan J., Hu Z., Su Z., Yang M.-H. (2017). L-0-regularized intensity and gradient prior for deblurring text images and beyond. IEEE Trans. Pattern Anal. Mach. Intell..

[8] Fergus R., Singh B., Hertzmann A., Roweis S.T., Freeman W.T. (2006). Removing camera shake from a single photograph. ACM Trans. Graph..

[9] Levin A., Fergus R., Durand F., Freeman W.T. (2007). Image and depth from a conventional camera with a coded aperture. ACM Trans. Graph..

[10] Wang Y., Yang J., Yin W., Zhang Y. (2008). A New Alternating Minimization Algorithm for Total Variation Image Reconstruction. SIAM J. Imaging Sci..

[11] Krishnan D., Tay T., Fergus R. Blind deconvolution using a normalized sparsity measure. Proceedings of the IEEE Conference on Computer Vision and Pattern Recognition (CVPR).

[12] Parameswaran S., Deledalle C.-A., Denis L., Nguyen T.Q. (2018). Accelerating GMM-based patch priors for image restoration: Three ingredients for a 100x speed-up. IEEE Trans. Image Process..

[13] Dong W., Zhang K., Shi G., Li X. (2012). Nonlocally Centralized Sparse Representation for Image Restoration. IEEE Trans. Image Process..

[14] Zoran D., Weiss Y. From learning models of natural image patches to whole image restoration. Proceedings of the IEEE International Conference on Computer Vision (ICCV).

[15] Danielyan A., Katkovnik V., Egiazarian K. (2011). BM3D Frames and Variational Image Deblurring. IEEE Trans. Image Process..

[16] Michaeli T., Irani M. Blind Deblurring Using Internal Patch Recurrence. Proceedings of the European Conference on Computer Vision.

[17] Shan Q., Jia J., Agarwala A. (2008). High-quality motion deblurring from a single image. ACM Trans. Graph..

[18] Pan J., Sun D., Pfister H., Yang M. Blind image deblurring using dark channel prior. Proceedings of the IEEE Conference on Computer Vision and Pattern Recognition (CVPR).

[19] Chen L., Fang F., Wang T., Zhang G. Blind image deblurring with local maximum gradient prior. Proceedings of the IEEE Conference on Computer Vision and Pattern Recognition (CVPR).

[20] Yan Y., Ren W., Guo Y., Wang R., Cao X. Image deblurring via extreme channels prior. Proceedings of the IEEE Conference on Computer Vision and Pattern Recognition (CVPR).

[21] Tao X., Gao H., Shen X., Wang J., Jia J. Scale-recurrent network for deep image deblurring. Proceedings of the IEEE Conference on Computer Vision and Pattern Recognition (CVPR).

[22] Liu X., Ma Y., Shi Z., Chen J. Griddehazenet: Attention-based multi-scale network for image dehazing. Proceedings of the 2019 IEEE/CVF International Conference on Computer Vision (ICCV).

[23] Sun J., Cao W., Xu Z., Ponce J. Learning a convolutional neural network for non-uniform motion blur removal. Proceedings of the IEEE Conference on Computer Vision and Pattern Recognition (CVPR).

[24] Yan R., Shao L. (2016). Blind image blur estimation via deep learning. IEEE Trans. Image Process..

[25] Cheng S., Liu R., He Y., Fan X., Luo Z. (2020). Blind image deblurring via hybrid deep priors modeling. Neurocomputing.

[26] Zhang Y., Tian Y., Kong Y., Zhong B., Fu Y. (2020). Residual Dense Network for Image Restoration. IEEE Trans. Pattern Anal. Mach. Intell..

[27] Pan J., Dong J., Liu Y., Zhang J., Ren J., Tang J., Tai Y.W., Yang M.-H. (2020). Physics-Based Generative Adversarial Models for Image Restoration and Beyond. IEEE Trans. Pattern Anal. Mach. Intell..

[28] Fei X., Zhao J., Zhao H., Yun D., Zhang Y. (2017). Deblurring adaptive optics retinal images using deep convolutional neural networks. Biomed. Opt. Express.

[29] Zhang K., Zhang Y., Zhong Y., Ma L., Liu W., Li H., Huang Y., Du Y., Wang L. (2019). Adversarial Spatio-Temporal Learning for Video Deblurring. IEEE Trans. Image Process..

[30] Wu J., Yu X., Liu D., Chandraker M., Wang Z. DAVID: Dual-Attentional Video Deblurring. Proceedings of the 2020 IEEE Winter Conference on Applications of Computer Vision (WACV).

[31] Dong W., Wang P., Yin W., Shi G., Wu F., Lu X. (2019). Denoising Prior Driven Deep Neural Network for Image Restoration. IEEE Trans. Pattern Anal. Mach. Intell..

[32] Minaee S., Wang Y. (2019). An ADMM Approach to Masked Signal Decomposition Using Subspace Representation. IEEE Trans. Image Process..

[33] Hu J., Shen L., Albanie S., Sun G., Wu E. (2019). Squeeze-and-Excitation Networks. IEEE Trans. Pattern Anal. Mach. Intell..

[34] Zhao W., Zhao F., Wang N., Lu H. (2019). Defocus Blur Detection via Multi-Stream Bottom-Top-Bottom Network. IEEE Trans. Pattern Anal. Mach. Intell..

[35] Shan H., Zhang Y., Yang Q., Kruger U., Kalra M.K., Sun L., Cong W., Wang G. (2018). 3D Convolutional Encoder-Decoder Network for Low-Dose CT via Transfer Learning From a 2D Trained Network. IEEE Trans. Med Imaging.

[36] Nair V., Hinton G.E. Rectified linear units improve restricted boltzmann machines. Proceedings of the International Conference on Machine Learning (ICML).

[37] Cao Y., Xu J., Lin S., Wei F., Hu H. GCNet: Non-Local Networks Meet Squeeze-Excitation Networks and Beyond. Proceedings of the 2019 IEEE/CVF International Conference on Computer Vision Workshop (ICCVW).

[38] Johnson J., Alahi A., Li F.-F. Perceptual Losses for Real-Time Style Transfer and Super-Resolution. Proceedings of the European Conference on Computer Vision.

[39] Kingma D.P., Ba L.J. Adam: A method for stochastic optimization. Proceedings of the International Conference on Learning Representations (ICLR).

[40] Nah S., Baik S., Hong S., Moon G., Son S., Timofte R., Lee K.M. NTIRE 2019 Challenge on Video Deblurring and Super-Resolution: Dataset and Study. Proceedings of the 2019 IEEE/CVF Conference on Computer Vision and Pattern Recognition Workshops (CVPRW).

[41] He K., Zhang X., Ren S., Sun J. Delving Deep into Rectifiers: Surpassing Human-Level Performance on ImageNet Classification. Proceedings of the 2015 IEEE International Conference on Computer Vision (ICCV).

[42] Nah S., Kim T.H., Lee K.M. Deep Multi-scale Convolutional Neural Network for Dynamic Scene Deblurring. Proceedings of the 2017 IEEE Conference on Computer Vision and Pattern Recognition (CVPR).

[43] Badri H., Yahia H., Aboutajdine D. (2016). Low-Rankness Transfer for Realistic Denoising. IEEE Trans. Image Process..

